# Parental Traits Associated with Adherence to the Mediterranean Diet in Children and Adolescents in Croatia: A Cross-Sectional Study

**DOI:** 10.3390/nu14132598

**Published:** 2022-06-23

**Authors:** Ivana Franić, Petra Boljat, Endica Radić Hozo, Ante Burger, Antonela Matana

**Affiliations:** 1 The University Department of Health Studies, University of Split, Ruđera Boškovića 35, 21000 Split, Croatia; ivana.zorica@ozs.unist.hr (I.F.); erhozo@ozs.unist.hr (E.R.H.); anteburger@gmail.com (A.B.); 2 Elementary School Žnjan-Pazdigrad, Pazdigradska 1, 21000 Split, Croatia; petraa.boljat@gmail.com

**Keywords:** Mediterranean diet, children, adolescents, parents, father, Croatia

## Abstract

The Mediterranean diet (MD) is known to be one of the healthiest dietary patterns. Despite the significance of a healthful diet during the early stage of life, data for young individuals indicate that nutrition problems are common. This cross-sectional study aimed to determine parental factors associated with MD adherence in children and adolescents living in the Mediterranean region in Croatia. In total, 2623 children aged 2 to 18 years and their parents participated in this study. Data were collected during the period from September 2021 to February 2022 by using an anonymous questionnaire. We used KIDMED and MEDAS questionnaires for assessing MD adherence in young individuals and their parents, respectively. To assess the association of children’s MD adherence categories with the parental predictors, we performed multivariate multinomial logistic regression. Results showed that the children of parents with a low MD adherence are much more likely to have poor MD adherence than good (OR = 47.54 (95% C.I 18.24, 123.87), *p* < 0.001) or average (OR = 5.64 (95% C.I 3.70, 8.6), *p* < 0.001) MD adherence. Further, children of fathers with higher BMI (OR = 1.035 (95% C.I 1.0, 1.071)) and those who do not live with both parents (OR = 1.703 (95% C.I 0.994, 2.916), *p* = 0.053) are more likely to have poor MD adherence than good MD adherence. These results indicate that interventions focusing on enhancing the quality of both parents’ diets could effectively improve their children’s eating habits.

## 1. Introduction

The Mediterranean diet (MD) is a dietary pattern that is characterized by the daily intake of fruit, vegetables, whole grains, nuts, seeds, and olive oil, and weekly intake of fish, dairy, and legumes, alongside low consumption of meat, eggs, and sweets [1]. It was widely reported to be a model of healthy eating for its contribution to beneficial health status and a better quality of life [2]. Evidence based on the reviews and meta-analyses suggests that adherence to the MD is inversely associated with the risk of cardiovascular disease, cancer, diabetes type 2, obesity, and improves cognitive health [3,4,5,6,7,8].

There is evidence that healthy lifestyle habits are established early in life and show long-term stability, making childhood and adolescence particularly important for the adoption and maintenance of healthy habits [9]. Despite the significance of a healthful diet during this stage of life, data for MD adherence largely varied within the European countries for both children and adolescents [10,11,12,13,14,15,16,17,18,19]. Poor MD adherence was recorded in 37% of Cypriot, 32.8% of Italian, and 14.9% of Greek children [10,11,12]. The best MD adherence was recorded among Spanish children, i.e., only 1.6% of 8–10-year-olds had poor MD adherence [13]. Few cross-sectional studies have been performed on Greek adolescents whose results indicated high prevalence (from 41.9% to 62.8%) of poor MD adherence [16,17,18]. Furthermore, poor MD adherence was identified in 39.6% of Italian adolescents [14]. Better results were recorded in Portugal and Spain, i.e., 7.2% of Portuguese adolescents and 4.7% of Spanish adolescents reported poor MD adherence [15,19]. Data for young populations (from kindergarten to college age) in Croatia indicate that nutrition problems are common [20,21,22,23]. In fact, in a recently published study conducted by our research team, it was shown that the prevalence rate of poor adherence to the MD in a sample of young individuals aged 2 to 24 years was 19.2%. It was also shown that the prevalence of poor nutrition increased with the higher educational stage, i.e., the lowest prevalence of poor MD adherence was observed for children enrolled in kindergartens (11.3%), then for primary school children (19.6% for 1st–4th grades and 16.8% for 5th–8th grades), followed by children from secondary schools (25.7%), while the highest prevalence of poor MD adherence was observed among college students (39.3%) [23].

The family is the most important environment for childhood development and learning, and parents play a crucial role in the development of the food preferences of their children [24,25]. Previous cross-sectional studies have shown that parental education, occupation and family income, as well as parents’ health knowledge and attention to health issues, are significant determinants of their children’s diet quality [12,24,26,27]. In the study performed by Arcila-Agudelo et al., it was shown that the mother’s education level, but not the father’s, was positively associated with the degree of adherence to the MD [28]. Other studies also showed that maternal educational level is positively associated with MD adherence [11,29]. The results of a longitudinal Australian study showed that children of mothers who were full-time employed at age 1 year had a below-average dietary quality score at age 14 years [30]. Furthermore, a recently published review also provided evidence that children of employed mothers had poorer dietary habits [31]. A large cross-sectional study performed in eight European countries (including Austria, Belgium, France, Germany, Sweden, Greece, Italy, and Spain) showed that children’s diet quality is influenced by socioeconomic and geographical factors [32]. Parental education level was found to be positively correlated with adolescents’ diet quality in northern Europe but not in southern Europe, while parental occupation level was positively correlated with adolescents’ diet quality in both northern and southern Europe [32]. There is evidence that adherence to MD among children differs according to the place they live (i.e., rural vs. urban), type of school attended and the child’s nationality [15,27]. Furthermore, the available literature suggests that family meals are associated with higher intakes of fruit, vegetables, calcium-rich foods, and key nutrients (e.g., calcium, iron, and folate), and a lower intake of soft drinks [33,34]. The food preferences of the parents are associated with their children’s eating behaviors [35,36,37]. Additionally, it was shown that irregular breakfast habits in children were associated with mothers being overweight or obese [38]. Despite published research on the association between parental traits and dietary habits in children, little research has been conducted on the parental traits associated with the MD exclusively, and there is a particular lack of research focusing on the association between parental MD adherence and children’s MD adherence. To the best of our knowledge, only one study on the association between parental and children’s MD adherence was performed. That is a cross-sectional study carried out on the 70 preschool children and their mothers from North Cyprus in which it was shown that the adherence of mothers to the MD positively correlated with the adherence to the MD in children [39]. Additionally, to our knowledge, there are no prior studies performed on the young Croatian population on the association of parental traits and children’s dietary habits.

Therefore, the aim of this study was to identify parental factors associated with MD adherence in a large sample of 2623 children and adolescents originating from the Mediterranean region in Croatia. This is the most comprehensive study performed so far on the association between parental and children’s MD adherence and the first one performed on the young Croatian population. Additionally, this study adds to the existing knowledge on the parental traits associated with MD adherence.

## 2. Materials and Methods

### 2.1. Study Design

This cross-sectional survey-based study was carried out in the Mediterranean region of Croatia (including regions of Istria, Kvarner, Dalmatia, Dubrovnik area, and the Adriatic Islands) during the period from September 2021 to February 2022. A total of 2623 children and adolescents, aged from 2 to 18 years, and their parents (aged from 23 to 70 years), were included in the study. We classified young participants into four groups in order to correspond to the Croatian educational system: (i) kindergartens, (ii) primary schools (1st–4th grade), (iii) primary schools (5th–8th grade), and (iv) secondary schools. The study was performed according to the principles of the latest Helsinki declaration and was approved by the Ethics Committee of the University Department of Health Studies, University of Split (Class 001-01/21-01/01, reg. no.: 2181-228-103/1-21-22). Participation in this study was voluntary and all subjects consented to participate by submitting the completed questionnaire.

### 2.2. Questionnaire

We sent an e-mail invitation to all kindergartens, primary and secondary schools located in the Mediterranean region of Croatia to participate in the research. The e-mail sent contained a description and purpose of the study and a link to the questionnaire used in the research. The questionnaire was created using the online program Google Forms. The representatives of the institutions forwarded the questionnaire to the parents via WhatsApp groups or emails. Additionally, in some institutions, the questionnaires were distributed in paper form since that was requested by the representatives of the institutions. The data from paper-based questionnaires were entered manually into the Microsoft Excel database. The expected time to complete the survey was about 10 min. For this study, the child’s parent (mother or father) completed the questionnaire for themselves and their children. Questionnaire was filled in by 2421 (92.3%) mothers and 202 (7.7%) fathers. Only one child per household was included in the study. We excluded children younger than two years. The survey included four sections. The first section was the general information questionnaire which assessed general data about the children and adolescents such as gender, age, type of study program, year of attendance, as well as weight and height. Weight and height were reported by the child’s parent who completed the questionnaire. Body mass index (BMI) was calculated by dividing weight in kilograms by the square of height in meters in order to calculate the BMI-for-age percentiles using the CDC growth charts [40]. Subsequently, the participants were grouped into four categories: underweight (percentiles lower than 5th), normal weight (percentiles between 5th and 85th), overweight (percentiles between 85th and 95th), and obese (percentiles ≥ 95th).

In the second part of the questionnaire, we used the KIDMED test (Mediterranean Diet Quality Index for children and adolescents) to assess the degree of adherence to the MD [41]. KIDMED is a questionnaire consisting of 16 dichotomous (yes or no) items, with 4 questions indicating a negative connotation with respect to MD (including consumption of fast food, baked goods, sweets, and skipping breakfast), and 12 questions with a positive connotation (consumption of fruits, vegetables, fish, pulses, pasta or rice, nuts, oil, cereals, dairy products, and yogurt). Questions with a negative connotation were assigned a value of −1, and those with a positive connotation were attributed a value of +1. The total score ranges between −4 to 12. Subjects with KIDMED scores ≤3 were considered to have a low MD adherence; those with scores 4–7 had average MD adherence, and those with scores ≥8 had a good MD adherence. The instrument was previously adapted for the Croatian language and tested for reliability and validity [20].

The third section consisted of questions related to the sociodemographic characteristics of parents. Information about age, educational level, weight (in kg), and height (in cm) was collected separately for each parent. Weight and height of both parents were reported by the parent who completed the questionnaire. BMI was calculated based on the reported weight and height. Parental employment status (with three possible answers: both parents employed, both parents unemployed, one parent employed and the other unemployed), as well as number of children, household size, and information on whether the child lives with both parents were recorded. Additionally, the financial status of the family, as assessed with a question: “Do finances limit your family in food choices?” (yes or no), was also recorded.

In the last section, the degree of adherence to the MD for parents was evaluated using the 14-item Mediterranean Diet Adherence Screener (MEDAS) questionnaire [42]. Each of the 14 items is scored 1 or 0, depending on whether participants adhere to each MEDAS component or not. The final MEDAS score can range between 0 and 14. For categorization of the adherence to the MD, the following criteria were applied: weak adherence, ≤5; moderate adherence, 6–9; good adherence, ≥10 [42]. The instrument was previously translated into the Croatian language and tested for reliability [43].

### 2.3. Statistical Analysis

Normality of distribution was tested using the Kolmogorov–Smirnov test. All categorical variables were presented using absolute numbers and percentages, and numerical variables were presented with medians and interquartile ranges (IQR) due to the non-normal distribution of the data. Differences between groups for categorical variables were analyzed with a Chi-square test and for numerical variables with Kruskal–Wallis tests. We also performed multivariate multinomial logistic regression in order to assess the association of children’s MD adherence categories with the odds ratios of parental predictors that were significant in univariate models (including father’s age, mother’s and father’s BMI, mother’s and father’s educational level, information whether the child lives with both parents, the financial status of the family and MEDAS categories). In our recently published study [23], we showed that adherence to the MD in children is associated with the stage of children’s education; therefore, we included children’s educational stage as a covariate in the model. *p*-values of less than 0.05 were considered statistically significant. Statistical analysis was carried out using Statistical Package Software for Social Science, version 28 (SPSS Inc., Chicago, IL, USA).

## 3. Results

A total of 2623 children and adolescents and their parents participated in this study. The basic characteristics of the study participants are presented in Table 1.

In the total sample of children, the median KIDMED index score was six (IQR: 3). The degree of adherence to the MD was poor in 17.7%, average in 61.7%, and good in 20.7% of the study participants. For parents’ adherence to the MD, the median MEDAS score was seven (IQR: 3). The majority of the parents had moderate MD adherence (66%), while 27.4% had low and only 6.6% had high MD adherence.

To assess the relationships between parental socio-demographic characteristics and MD adherence and children’s MD adherence, data were processed firstly by univariate analyses aimed at identifying dependence between the variables investigated. The results are shown in Table S1. Results revealed that children of less-educated mothers (*p* < 0.001) and fathers (*p* < 0.001), those who do not live with both parents (*p* = 0.018), and those whose families are financially limited in their food choices (*p* = 0.008), more frequently have low MD adherence. Higher father and mother BMI index (*p* = 0.003, *p* = 0.003, respectively), as well as a higher father’s age (*p* = 0.013), were associated with a higher frequency of low MD adherence. A significant association was also observed between the parental and children’s MD adherence; children of parents with low adherence more frequently also had low MD adherence (*p* < 0.001). Variables such as the mother’s age (*p* = 0.155), employment status (*p* = 0.272), number of children (*p* = 0.680), and household size (*p* = 0.158) did not show a significant association with the children’s MD adherence.

Once the dependence was determined, a multivariate multinomial logistic regression was carried out for children’s MD adherence with eight significant predictors in the univariate model (*p* < 0.001, Nagelkerke R^2^ = 0.130, correct prediction rate 61.4%). The results of the multivariate model are shown in Table 2. Parental MD adherence seems to be the most important predictor of children’s MD adherence, as children of parents with low MD adherence are much more likely to have poor MD adherence than good (OR = 47.54 (95% C.I 18.24, 123.87), *p* < 0.001) or average (OR = 5.64 (95% C.I 3.70, 8.6), *p* < 0.001) MD adherence. Furthermore, children of fathers with a higher BMI (OR = 1.035 (95% C.I 1.0, 1.071)) are more likely to have poor MD adherence than good MD adherence. For children who do not live with both parents, a borderline significance was observed for having higher odds for MD adherence than good MD adherence (OR = 1.703 (95% C.I 0.994, 2.916), *p* = 0.053).

## 4. Discussion

The primary purpose of this study was to determine parental factors associated with adherence to the MD in children and adolescents from the Mediterranean region in Croatia. To the best of our knowledge, this is the first study in the Croatian population that examined parental factors associated with diet quality in youth. We identified several predictors of children’s adherence to the MD, including parental MD adherence, father’s BMI, and living with both parents.

Parental MD adherence seems to be the most significant predictor of children’s MD adherence. Our study showed that children of parents with low MD adherence are much more likely to have poor MD adherence than good or average MD adherence. Although convincing evidence supports a relationship between child–parent dietary intakes, direct comparisons to current literature are difficult because, to the best of our knowledge, only one study with a small sample size has specifically examined the association between parental and children’s MD adherence. That is a cross-sectional study performed on the 70 preschool children and their mothers from North Cyprus in which it was shown that the adherence of mothers to the MD is positively associated with the adherence to the MD in children [39]. However, our results are not surprising, as it is known that parental influence is very important in acquiring healthy eating and lifestyle habits. Previous studies consistently demonstrate that the home food environment and parental dietary habits are determinants of children’s dietary intake [44,45]. Several cross-sectional studies reported a positive association between parental and children’s intake of some of MD’s beneficial compounds [46,47]. More precisely, recently published studies showed that fathers’ fruit and vegetable intake was positively associated with children’s intake of these foods [47] as well as that the highest percentage of children who always consumed breakfast were those whose mothers always consumed it [46]. Additionally, the previous research suggests that maternal consumption of healthy food (e.g., fruit, vegetables, dairy, and cereals,) and unhealthy foods (e.g., fats, snack foods, and oils) is associated with a child’s higher intake of the same foods [48]. In this context, parental dietary habits are likely to play an important role in shaping children’s dietary habits, probably through positive role-modeling and by controlling the availability and accessibility of different types of food at home. Differently designed studies (i.e., cross-sectional study, focus-group discussion) confirmed that the availability of healthy food and the absence of unhealthy alternatives influence children’s intake of those foods [49,50,51]. On the contrary, unhealthy eating patterns in parents could harm establishing healthy eating patterns in children. Therefore, interventions focusing on improving the quality of both parents’ diets could effectively improve their children’s eating habits.

Another important finding of this study is an association between fathers’ BMI and children’s MD adherence. A few studies designed differently (i.e., randomized controlled trials and qualitative studies) demonstrated that fathers play a crucial role in shaping their children’s dietary habits and level of physical activity, which are significantly associated with a child’s weight status [52,53,54]. Previous research demonstrated that the fathers’ influence on the child’s food choices was highest for the sweet and fats food group (e.g., sugar and sweets, unhealthy fats and oils, unhealthy beverages, and unhealthy milk and dairy products) [55]. Likewise, another study also revealed a positive association between fast food intake among children and adolescents and the father’s increased BMI [56]. Hall and colleagues reported strong associations between father and child intake of fruit and some nutrient-poor foods, even after controlling for maternal diet [57]. Additionally, fathers’ frequency of use of fast-food restaurants and perceptions of dinner as an important family habit was associated with child fast-food consumption [58]. Another study reported stronger associations between father–child macronutrients and energy intakes compared to the mother–child pairs [59]. Additionally, a large study revealed that children with an obese father but a healthy-weight mother were 15 times more likely to be obese than children with healthy-weight parents [60]. On the contrary, having an obese mother but a healthy-weight father did not increase the risk of childhood obesity [61]. This suggests that, apart from mothers, fathers should be considered as potential agents for the implementation of positive feeding practices in children.

Additionally, our study reported a better MD adherence among those children and adolescents that live with both parents. This result is in line with the recently performed study in Turkish preschool children (4–6 years) in which it was shown that MD adherence of children whose parents are together was significantly higher than in children whose parents are separated [62]. Similarly, it was shown that there might be an increased risk of being overweight or obese in children whose parents are separated [63]. The association between the marital status of the parents and the child’s dietary habits was also confirmed in previous studies [64]. Namely, it was shown that children with divorced parents more often consume sugary drinks and skip breakfast meals than children living with both parents [65], while children living with both parents more often consume fruits and milk, and dairy products [64].

Interestingly, we did not find a significant association between parents’ education level, employment status and the financial status of the family with the children’s MD adherence in a multivariate analysis. One possible explanation may be that unemployed parents usually have more time to spend on preparing and eating healthy meals with their children, but also a lower budget for buying healthy groceries and cooking equipment when compared to employed parents [66]. Additionally, it is possible that highly educated parents do not necessarily have high nutritional knowledge, as recorded in the study performed at the Kent State University in which it was shown that highly educated mothers had extremely low nutrition knowledge [67]. Contrary to our findings, some other cross-sectional studies demonstrated a positive association between household education and the quality of children’s diet [68,69,70]. Additionally, few cross-sectional studies showed that the percentage of children with low adherence to the MD decreased with increased maternal education level [11,12,26]. In addition, several studies found a positive association between household income and adherence to the MD [15,41]. Moreover, results of the study performed by Rosso et. al in Italian adolescents identified high socioeconomic status as one of the major predictors of high adherence to the MD [27]. However, findings from the HELENA study, which was performed on adolescents from eight European countries, showed that parental education level was positively correlated with diet quality in northern Europe but not in southern Europe, while parental occupation level was positively correlated with adolescents’ diet quality in both northern and southern Europe [32]. A possible explanation for differences between our study and previous research might be due to population differences, the definition of financial status, categorization of educational levels, and statistical methods employed in the analyses.

There are several limitations to this study that are worth mentioning. The main limitation of the study is the cross-sectional design, and therefore, a causal link cannot be established. Another limitation of this study is that MD adherence variables observed were not objectively measured but were reported by the parents of the children, therefore, there is a possibility of misreporting and data bias. Weight and height were also parent-reported which could potentially lead to an error in the reports. However, it was shown that parents are relatively accurate in reporting their child’s height and weight, indicating that height and weight reported by the parents could be used as a valid method of collecting child anthropometric data [71]. Additionally, we did not collect data on some possibly important confounding variables that could influence the observed associations (i.e., full-time or part-time employment of parents). Another limitation of our study is that we collected data for dietary habits only for one parent. However, our study has also many strengths, including the size of the sample of participants from an entire Mediterranean region of Croatia and within a wide age range, as well as the use of reliable and validated scales. This is the first study conducted so far on MD adherence in the youth population in Croatia where results have undoubtedly improved the existing knowledge on predictors of MD adherence among children and the youth.

## 5. Conclusions

To conclude, this study expands upon the existing body of literature that explores parental factors associated with children’s MD adherence. Results support the evidence that evaluation and correction of poor dietary habits of parents might have a crucial effect on child’s dietary habits, and therefore, public health strategies should focus on improving parental eating behavior rather than just educating parents on healthy eating patterns for their children. Our results also highlighted the important role of fathers’ obesity status on their children’s MD adherence and, therefore, interventions designed to improve diet quality in children may be enhanced by including fathers. Finally, living with both parents was also associated with better diet quality in children.

## Figures and Tables

**Table 1:** Basic characteristics of the study participants.

Variable	Descriptive Statistics
Children and Adolescents	
Gender	*n* (%)
Females	1364 (52%)
Males	1253 (47.8%)
Age, median (interquartile range)	10.0 (6.0)
BMI Classification	*n* (%)
Underweight	198 (7.5%)
Normal weight	1795 (68.4%)
Overweight	335 (12.8%)
Obese	165 (6.3%)
Educational Stage	*n* (%)
Kindergarten	493 (18.8%)
Primary school (1st–4th grade)	964 (36.8%)
Primary school (5th–8th grade)	813 (31%)
Secondary school	348 (13.3%)
Parents	
Mother’s age, median (interquartile range)	39.0 (7.0)
Father’s age, median (interquartile range)	42.0 (8.0)
Mother’s BMI, median (interquartile range)	22.59 (4.03)
Father’s BMI, median (interquartile range)	26.87 (4.25)
Mother’s Educational Level	*n* (%)
Primary school	24 (0.9%)
High school	1200 (45.7%)
Bachelor degree	281 (10.7%)
Master’s degree	1045 (39.8%)
Ph.D. degree	47 (1.8%)
Father’s Educational Level	*n* (%)
Primary school	43 (1.6%)
High school	1510 (57.6%)
Bachelor degree	256 (9.8%)
Master’s degree	688 (26.2%)
Ph.D. degree	51 (1.9%)

**Table 2:** Results of the multinomial logistic regression with categories of children MD adherence as a dependent variable and parental traits as independent variables.

Predictors	Poor MD Adherence		Average MD Adherence	
	OR (95% CI) ^1^	*p*-Value	OR (95% CI) ^1^	*p*-Value
Father’s Age	1.009 (0.990, 1.029)	0.361	1.010 (0.992, 1.029)	0.267
Mother’s BMI	1.034 (0.995, 1.075)	0.093	1.008 (0.978, 1.040)	0.599
Father’s BMI	1.035 (1.0, 1.071)	0.048	1.024 (0.995, 1.054)	0.105
Mother’s Education Level				
Primary school	2.331 (0.29, 18.723)	0.426	0.554 (0.131, 2.336)	0.421
Secondary school	2.685 (0.534, 13.508)	0.231	0.647 (0.284, 1.474)	0.300
Bachelor degree	3.167 (0.611, 16.429)	0.170	0.636 (0.270, 1.501)	0.302
Master degree	2.063 (0.414, 10.273)	0.377	0.594 (0.265, 1.332)	0.206
Ph.D. degree	-	-	-	-
Father’s Education Level				
Primary school	1.032 (0.266, 4.003)	0.963	0.388 (0.121, 1.247)	0.112
Secondary school	0.857 (0.306, 2.398)	0.768	0.880 (0.402, 1.926)	0.749
Bachelor degree	0.888 (0.296, 2.665)	0.832	1.046 (0.456, 2.403)	0.915
Master degree	0.645 (0.230, 1.809)	0.404	0.799 (0.366, 1.744)	0.572
Ph.D. degree	-	-	-	-
Living with Both Parents				
No	1.703 (0.994, 2.916)	0.053	1.209 (0.768, 1.905)	0.413
Yes	-	-	-	-
Do Finances Limit Your Family in Food Choices?				
No	1.029 (0.732, 1.447)	0.869	1.096 (0.836, 1.438)	0.506
Yes	-	-	-	-
Medas Categories				
Low	47.537 (18.243, 123.874)	<0.001	5.641 (3.703, 8.595)	<0.001
Moderate	8.080 (3.201, 20.397)	<0.001	2.259 (1.611, 3.168)	<0.001
High	-	-	-	-

^1^ Odds ratios (OR) were calculated by multivariate multinomial logistic regression with good MD adherence as the reference category in the dependent variable.

## Data Availability

Raw data can be obtained from the corresponding author via e-mail: antonela.matana@gmail.com.

## Supplementary Materials

The following are available online at https://www.mdpi.com/article/10.3390/nu14132598/s1, Table S1: Results of univariate analyses for the relationships between parental socio-demographic characteristics and MD adherence and children’s MD adherence.

## References

[1] Bach-Faig A., Berry E.M., Lairon D., Reguant J., Trichopoulou A., Dernini S., Medina F.X., Battino M., Belahsen R., Miranda G. (2011). Mediterranean diet pyramid today. Science and cultural updates. Public Health Nutr..

[2] Sofi F., Macchi C., Abbate R., Gensini G.F., Casini A. (2014). Mediterranean diet and health status: An updated meta-analysis and a proposal for a literature-based adherence score. Public Health Nutr..

[3] Esposito K., Maiorino M.I., Bellastella G., Chiodini P., Panagiotakos D., Giugliano D. (2015). A journey into a Mediterranean diet and type 2 diabetes: A systematic review with meta-analyses. BMJ Open.

[4] Mattioli A.V., Palmiero P., Manfrini O., Puddu P.E., Nodari S., Dei Cas A., Mercuro G., Scrutinio D., Palermo P., Sciomer S. (2017). Mediterranean diet impact on cardiovascular diseases: A narrative review. J. Cardiovasc. Med..

[5] Schwingshackl L., Schwedhelm C., Galbete C., Hoffmann G. (2017). Adherence to Mediterranean Diet and Risk of Cancer: An Updated Systematic Review and Meta-Analysis. Nutrients.

[6] Grosso G., Marventano S., Yang J., Micek A., Pajak A., Scalfi L., Galvano F., Kales S.N. (2017). A comprehensive meta-analysis on evidence of Mediterranean diet and cardiovascular disease: Are individual components equal?. Crit. Rev. Food Sci. Nutr..

[7] Liyanage T., Ninomiya T., Wang A., Neal B., Jun M., Wong M.G., Jardine M., Hillis G.S., Perkovic V. (2016). Effects of the Mediterranean Diet on Cardiovascular Outcomes-A Systematic Review and Meta-Analysis. PLoS ONE.

[8] Martin-Pelaez S., Fito M., Castaner O. (2020). Mediterranean Diet Effects on Type 2 Diabetes Prevention, Disease Progression, and Related Mechanisms. A Review. Nutrients.

[9] Mikkila V., Rasanen L., Raitakari O.T., Pietinen P., Viikari J. (2005). Consistent dietary patterns identified from childhood to adulthood: The cardiovascular risk in Young Finns Study. Br. J. Nutr..

[10] Lazarou C., Panagiotakos D.B., Matalas A.L. (2009). Level of adherence to the Mediterranean diet among children from Cyprus: The CYKIDS study. Public Health Nutr..

[11] Roccaldo R., Censi L., D’Addezio L., Toti E., Martone D., D’Addesa D., Cernigliaro A., the ZOOM8 Study Group (2014). Adherence to the Mediterranean diet in Italian school children (The ZOOM8 Study). Int. J. Food Sci. Nutr..

[12] Kontogianni M.D., Vidra N., Farmaki A.E., Koinaki S., Belogianni K., Sofrona S., Magkanari F., Yannakoulia M. (2008). Adherence rates to the Mediterranean diet are low in a representative sample of Greek children and adolescents. J. Nutr..

[13] Mariscal-Arcas M., Rivas A., Velasco J., Ortega M., Caballero A.M., Olea-Serrano F. (2009). Evaluation of the Mediterranean Diet Quality Index (KIDMED) in children and adolescents in Southern Spain. Public Health Nutr..

[14] Noale M., Nardi M., Limongi F., Siviero P., Caregaro L., Crepaldi G., Maggi S., Mediterranean Diet Foundation Study Group (2014). Adolescents in southern regions of Italy adhere to the Mediterranean diet more than those in the northern regions. Nutr. Res..

[15] Arriscado D., Muros J.J., Zabala M., Dalmau J.M. (2014). Factors associated with low adherence to a Mediterranean diet in healthy children in northern Spain. Appetite.

[16] Mazaraki A., Tsioufis C., Dimitriadis K., Tsiachris D., Stefanadi E., Zampelas A., Richter D., Mariolis A., Panagiotakos D., Tousoulis D. (2011). Adherence to the Mediterranean diet and albuminuria levels in Greek adolescents: Data from the Leontio Lyceum ALbuminuria (3L study). Eur. J. Clin. Nutr..

[17] Magriplis E., Farajian P., Pounis G.D., Risvas G., Panagiotakos D.B., Zampelas A. (2011). High sodium intake of children through ‘hidden’ food sources and its association with the Mediterranean diet: The GRECO study. J. Hypertens..

[18] Bargiota A., Pelekanou M., Tsitouras A., Koukoulis G.N. (2013). Eating habits and factors affecting food choice of adolescents living in rural areas. Hormones.

[19] Da Rocha Leal F.M., De Oliveira B.M.P.M., Pereira S.S.R. (2011). Relationship between cooking habits and skills and Mediterranean diet in a sample of Portuguese adolescents. Perspect. Public Health.

[20] Stefan L., Prosoli R., Juranko D., Cule M., Milinovic I., Novak D., Sporis G. (2017). The Reliability of the Mediterranean Diet Quality Index (KIDMED) Questionnaire. Nutrients.

[21] Obradovic Salcin L., Karin Z., Miljanovic Damjanovic V., Ostojic M., Vrdoljak A., Gilic B., Sekulic D., Lang-Morovic M., Markic J., Sajber D. (2019). Physical Activity, Body Mass, and Adherence to the Mediterranean Diet in Preschool Children: A Cross-Sectional Analysis in the Split-Dalmatia County (Croatia). Int. J. Environ. Res. Public Health.

[22] Bučan Nenadić D., Kolak E., Selak M., Smoljo M., Radić J., Vučković M., Dropuljić B., Pijerov T., Babić Cikoš D. (2021). Anthropometric Parameters and Mediterranean Diet Adherence in Preschool Children in Split-Dalmatia County, Croatia—Are They Related?. Nutrients.

[23] Matana A., Franic I., Radic Hozo E., Burger A., Boljat P. (2022). Adherence to the Mediterranean Diet among Children and Youth in the Mediterranean Region in Croatia: A Comparative Study. Nutrients.

[24] Wu J.C.-L. (2018). Parental work characteristics and diet quality among pre-school children in dual-parent households: Results from a population-based cohort in Taiwan. Public Health Nutr..

[25] Wardle J., Carnell S., Cooke L. (2005). Parental control over feeding and children’s fruit and vegetable intake: How are they related?. J. Am. Diet. Assoc..

[26] Schroder H., Mendez M.A., Ribas-Barba L., Covas M.I., Serra-Majem L. (2010). Mediterranean diet and waist circumference in a representative national sample of young Spaniards. Int. J. Pediatr. Obes..

[27] Grosso G., Marventano S., Buscemi S., Scuderi A., Matalone M., Platania A., Giorgianni G., Rametta S., Nolfo F., Galvano F. (2013). Factors associated with adherence to the Mediterranean diet among adolescents living in Sicily, Southern Italy. Nutrients.

[28] Arcila-Agudelo A.M., Ferrer-Svoboda C., Torres-Fernandez T., Farran-Codina A. (2019). Determinants of Adherence to Healthy Eating Patterns in a Population of Children and Adolescents: Evidence on the Mediterranean Diet in the City of Mataro (Catalonia, Spain). Nutrients.

[29] Buja A., Grotto G., Brocadello F., Sperotto M., Baldo V. (2020). Primary school children and nutrition: Lifestyles and behavioral traits associated with a poor-to-moderate adherence to the Mediterranean diet. A cross-sectional study. Eur. J. Pediatr..

[30] Li J., O’Sullivan T., Johnson S., Stanley F., Oddy W. (2012). Maternal work hours in early to middle childhood link to later adolescent diet quality. Public Health Nutr..

[31] Afrin S., Mullens A.B., Chakrabarty S., Bhowmik L., Biddle S.J.H. (2021). Dietary habits, physical activity, and sedentary behaviour of children of employed mothers: A systematic review. Prev. Med. Rep..

[32] Beghin L., Dauchet L., De Vriendt T., Cuenca-Garcia M., Manios Y., Toti E., Plada M., Widhalm K., Repasy J., Huybrechts I. (2014). Influence of parental socio-economic status on diet quality of European adolescents: Results from the HELENA study. Br. J. Nutr..

[33] Neumark-Sztainer D., Hannan P.J., Story M., Croll J., Perry C. (2003). Family meal patterns: Associations with sociodemographic characteristics and improved dietary intake among adolescents. J. Am. Diet. Assoc..

[34] Gillman M.W., Rifas-Shiman S.L., Frazier A.L., Rockett H.R., Camargo C.A., Field A.E., Berkey C.S., Colditz G.A. (2000). Family dinner and diet quality among older children and adolescents. Arch. Fam. Med..

[35] Grimm G.C., Harnack L., Story M. (2004). Factors associated with soft drink consumption in school-aged children. J. Am. Diet. Assoc..

[36] Scaglioni S., Salvioni M., Galimberti C. (2008). Influence of parental attitudes in the development of children eating behaviour. Br. J. Nutr..

[37] Sutherland L.A., Beavers D.P., Kupper L.L., Bernhardt A.M., Heatherton T., Dalton M.A. (2008). Like parent, like child: Child food and beverage choices during role playing. Arch. Pediatr. Adolesc. Med..

[38] Alsharairi N.A., Somerset S.M. (2016). Skipping breakfast in early childhood and its associations with maternal and child BMI: A study of 2-5-year-old Australian children. Eur. J. Clin. Nutr..

[39] Dayi T., Soykut G., Ozturk M., Yucecan S. (2021). Mothers and Children Adherence to the Mediterranean Diet: Evidence from a Mediterranean Country. Prog. Nutr..

[40] Children’s BMI Tool for Schools. https://www.cdc.gov/healthyweight/assessing/bmi/childrens_bmi/tool_for_schools.html.

[41] Serra-Majem L., Ribas L., Ngo J., Ortega R.M., Garcia A., Perez-Rodrigo C., Aranceta J. (2004). Food, youth and the mediterranean diet in Spain. Development of KIDMED, Mediterranean Diet Quality Index in children and adolescents. Public Health Nutr..

[42] Martinez-Gonzalez M.A., Garcia-Arellano A., Toledo E., Salas-Salvado J., Buil-Cosiales P., Corella D., Covas M.I., Schroder H., Aros F., Gomez-Gracia E. (2012). A 14-item Mediterranean diet assessment tool and obesity indexes among high-risk subjects: The PREDIMED trial. PLoS ONE.

[43] Marendic M., Polic N., Matek H., Orsulic L., Polasek O., Kolcic I. (2021). Mediterranean diet assessment challenges: Validation of the Croatian Version of the 14-item Mediterranean Diet Serving Score (MDSS) Questionnaire. PLoS ONE.

[44] Neumark-Sztainer D., Wall M., Perry C., Story M. (2003). Correlates of fruit and vegetable intake among adolescents. Findings from Project EAT. Prev. Med..

[45] O’Dea J.A. (2003). Why do kids eat healthful food? Perceived benefits of and barriers to healthful eating and physical activity among children and adolescents. J. Am. Diet. Assoc..

[46] Gimenez-Legarre N., Santaliestra-Pasias A.M., Cardon G., Imre R., Iotova V., Kivela J., Liatis S., Makrilakis K., Mavrogianni C., Milenkovic T. (2021). Cross-Sectional Associations between Mothers and Children’s Breakfast Routine-The Feel4Diabetes-Study. Nutrients.

[47] Papamichael M.M., Moschonis G., Mavrogianni C., Liatis S., Makrilakis K., Cardon G., De Vylder F., Kivela J., Flores-Barrantes P., Imre R. (2022). Fathers’ daily intake of fruit and vegetables is positively associated with children’s fruit and vegetable consumption patterns in Europe: The Feel4Diabetes Study. J. Hum. Nutr. Diet..

[48] Johnson L., van Jaarsveld C.H., Wardle J. (2011). Individual and family environment correlates differ for consumption of core and non-core foods in children. Br. J. Nutr..

[49] Nielsen S.J., Siega-Riz A.M., Popkin B.M. (2002). Trends in food locations and sources among adolescents and young adults. Prev. Med..

[50] Hanson N.I., Neumark-Sztainer D., Eisenberg M.E., Story M., Wall M. (2005). Associations between parental report of the home food environment and adolescent intakes of fruits, vegetables and dairy foods. Public Health Nutr..

[51] Beets M.W., Tilley F., Kyryliuk R., Weaver R.G., Moore J.B., Turner-McGrievy G. (2014). Children select unhealthy choices when given a choice among snack offerings. J. Acad. Nutr. Diet..

[52] Lloyd A.B., Lubans D.R., Plotnikoff R.C., Morgan P.J. (2015). Paternal Lifestyle-Related Parenting Practices Mediate Changes in Children’s Dietary and Physical Activity Behaviors: Findings from the Healthy Dads, Healthy Kids Community Randomized Controlled Trial. J. Phys. Act. Health.

[53] Lubans D.R., Morgan P.J., Collins C.E., Okely A.D., Burrows T., Callister R. (2012). Mediators of weight loss in the ‘Healthy Dads, Healthy Kids’ pilot study for overweight fathers. Int. J. Behav. Nutr. Phys. Act..

[54] Zahra J., Sebire S.J., Jago R. (2015). “He’s probably more Mr. sport than me”—A qualitative exploration of mothers’ perceptions of fathers’ role in their children’s physical activity. BMC Pediatr..

[55] Hebestreit A., Intemann T., Siani A., De Henauw S., Eiben G., Kourides Y.A., Kovacs E., Moreno L.A., Veidebaum T., Krogh V. (2017). Dietary Patterns of European Children and Their Parents in Association with Family Food Environment: Results from the I.Family Study. Nutrients.

[56] Qiu C., Hou M. (2020). Association between Food Preferences, Eating Behaviors and Socio-Demographic Factors, Physical Activity among Children and Adolescents: A Cross-Sectional Study. Nutrients.

[57] Hall L., Collins C.E., Morgan P.J., Burrows T.L., Lubans D.R., Callister R. (2011). Children’s intake of fruit and selected energy-dense nutrient-poor foods is associated with fathers’ intake. J. Am. Diet. Assoc..

[58] McIntosh A., Kubena K.S., Tolle G., Dean W., Kim M.-J., Jan J.-S., Anding J. (2011). Determinants of children’s use of and time spent in fast-food and full-service restaurants. J. Nutr. Educ. Behav..

[59] Vauthier J.M., Lluch A., Lecomte E., Artur Y., Herbeth B. (1996). Family resemblance in energy and macronutrient intakes: The Stanislas Family Study. Int. J. Epidemiol..

[60] Freeman E., Fletcher R., Collins C.E., Morgan P.J., Burrows T., Callister R. (2012). Preventing and treating childhood obesity: Time to target fathers. Int. J. Obes..

[61] Harrist A.W., Swindle T.M., Hubbs-Tait L., Topham G.L., Shriver L.H., Page M.C. (2016). The Social and Emotional Lives of Overweight, Obese, and Severely Obese Children. Child Dev..

[62] Sanlier N., Ulusoy H.G., Kocabas S., Celik B., Gobel P., Yilmaz S. (2021). Mediterranean Diet Adherence among Preschoolers and Its Association with Parents’ Beliefs, Attitudes, and Practices. Ecol. Food Nutr..

[63] Goisis A., Ozcan B., Van Kerm P. (2019). Do Children Carry the Weight of Divorce?. Demography.

[64] Baek Y.J., Paik H.Y., Shim J.E. (2014). Association between family structure and food group intake in children. Nutr. Res. Pract..

[65] Mauskopf S.S., O’Leary A.K., Banihashemi A., Weiner M., Cookston J.T. (2015). Divorce and eating behaviors: A 5-day within-subject study of preadolescent obesity risk. Child. Obes..

[66] Alsharairi N.A., Somerset S. (2018). Parental work status and children’s dietary consumption: Australian evidence. Int. J. Consum. Stud..

[67] Patel P.J. (2020). Differences between Nutritional Knowledge of Mothers of Preeschoolers and the Growth Status and Dietary Intake of Preschoolers. Master’s Thesis.

[68] Fernandez-Alvira J.M., Mouratidou T., Bammann K., Hebestreit A., Barba G., Sieri S., Reisch L., Eiben G., Hadjigeorgiou C., Kovacs E. (2013). Parental education and frequency of food consumption in European children: The IDEFICS study. Public Health Nutr..

[69] Riediger N.D., Shooshtari S., Moghadasian M.H. (2007). The influence of sociodemographic factors on patterns of fruit and vegetable consumption in Canadian adolescents. J. Am. Diet. Assoc..

[70] Sausenthaler S., Kompauer I., Mielck A., Borte M., Herbarth O., Schaaf B., von Berg A., Heinrich J. (2007). Impact of parental education and income inequality on children’s food intake. Public Health Nutr..

[71] Chai L.K., Collins C.E., May C., Holder C., Burrows T.L. (2019). Accuracy of Parent-Reported Child Height and Weight and Calculated Body Mass Index Compared with Objectively Measured Anthropometrics: Secondary Analysis of a Randomized Controlled Trial. J. Med. Internet Res..

