# Supplementary material for: Parental Traits Associated with Adherence to the Mediterranean Diet in Children and Adolescents in Croatia: A Cross-Sectional Study
Source: Nutrients. 2022 Jun 23;14(13):2598. doi: 10.3390/nu14132598 (PMC9268300; doi:10.3390/nu14132598)
Supplement: Supplementary file 1 [file nutrients-14-02598-s001.zip › nutrients-1757687-supplementary.pdf]

Table S1. Results of univariate analyses for the relationships between parental socio-demographic characteristics and MD adherence and children's MD adherence.

Variables	KIDMED categories			*p*-value
	Low	Average	High	
Mother's age, median (IQR)	40.0 (8.0)	39.0 (7.0)	39.0 (8.0)	0.155
Father's age, median (IQR)	43.0 (7.0)	42.0 (8.0)	42.0 (9.0)	0.013
Mother's BMI, median (IQR)	23.05 (4.75)	22.51 (3.93)	22.49 (3.85)	0.003
Father's BMI, median (IQR)	27.40 (4.44)	26.87 (4.22)	26.83 (4.22)	0.003
Mother's educational level, N (%)				<0.001
Primary school	7 (29.2)	13 (54.2)	4 (16.7)	
High school	253 (21.1)	720 (60.0)	227 (18.9)	
Bachelor degree	55 (19.6)	170 (60.5)	56 (19.9)	
Master's degree	137 (13.1)	664 (63.5)	244 (23.3)	
PhD degree	2 (4.3%)	37 (78.7%)	8 (17%)	
Father's educational level, N(%)				<0.001
Primary school	18 (41.9)	16 (37.2)	9 (20.9)	
High school	295 (19.5%)	920 (60.9)	295 (19.5)	
Bachelor degree	38 (14.8)	167 (65.2)	51 (19.9)	
Master's degree	85 (12.4)	437 (63.5)	166 (24.1)	
PhD degree	8 (15.7)	34 (66.7)	9 (17.6)	
Living with both parents				0.018
Yes	399 (16.9)	1467 (62.0)	499 (21.1)	
No	56 (24.1)	136 (58.6)	40 (17.2)	
Employment status				0.272
Both parents employed	341 (16.7)	1272 (62.2)	433 (21.2)	
One parent employed, the other unemployed	109 (21.2)	310 (60.3)	95 (18.5)	
Both parents unemployed	6 (19.4)	17 (548)	8 (25.8)	
Number of children				0.680
1	80 (18%)	269 (60.4%)	96 (21.6%)	
2	226 (16.9%)	847 (63.3%)	266 (19.9%)	
3	121 (18.8%)	389 (60.5%)	133 (20.7%)	
≥ 4	36 (18.4%)	113 (57.7%)	47 (24%)	
Household size				0.158
2	18 (25.4%)	41 (57.7)	12 (16.9)	
3	82 (18.5)	264 (59.6)	97 (21.9)	
4	206 (16.5)	804 (64.3)	241 (19.3)	
5	113 (17.8)	389 (61.2)	134 (21.1)	
6	27 (19.6)	73 (52.9)	38 (27.5)	
≥ 7	9 (15.5)	33 (56.9)	16 (27.6)	
Do finances limit your family in food choices?				0.008
Yes	121 (22.1%)	324 (59.1%)	103 (18.8%)	
No	336 (16.5%)	1271 (62.3%)	434 (21.3%)	
MEDAS categories for MD adherence				<0.001
Low	225 (31.3)	427 (59.4)	67 (9.3)	
Moderate	232 (13.4)	1098 (63.5)	400 (23.1)	
High	6 (3.4)	93 (53.4)	75 (43.1)	
